# Autophagy is required for the therapeutic effects of the NAD^+^ precursor nicotinamide in obesity-related heart failure with preserved ejection fraction

**DOI:** 10.1093/eurheartj/ehaf062

**Published:** 2025-02-25

**Authors:** Mahmoud Abdellatif, Francisco Vasques-Nóvoa, Viktoria Trummer-Herbst, Sylvère Durand, Franziska Koser, Moydul Islam, Jihoon Nah, Eun-Ah Sung, Ruli Feng, Fanny Aprahamian, Andreas Prokesch, Pablo Zardoya-Laguardia, Junichi Sadoshima, Abhinav Diwan, Wolfgang A Linke, João Pedro Ferreira, Guido Kroemer, Simon Sedej

**Affiliations:** Department of Cardiology, Medical University of Graz, Auenbruggerplatz 15, Graz 8036, Austria; Metabolomics and Cell Biology Platforms, Institut Gustave Roussy, 114 Rue Edouard Vaillant, Villejuif 94805, France; Centre de Recherche des Cordeliers, Equipe labellisée par la Ligue contre le cancer, Université de Paris, Sorbonne Université, INSERM U1138, Institut Universitaire de France, 15 Rue de l'École de Médecine, Paris 75006, France; BioTechMed-Graz, Mozartgasse 12/I, Graz 8010, Austria; Department of Internal Medicine, Centro Hospitalar Universitário de São João, 4200-319 Porto, Portugal; Department of Surgery and Physiology, Faculty of Medicine of the University of Porto, Cardiovascular R&D Center (UnIC@RISE), 4200-319 Porto, Portugal; Department of Cardiology, Medical University of Graz, Auenbruggerplatz 15, Graz 8036, Austria; Metabolomics and Cell Biology Platforms, Institut Gustave Roussy, 114 Rue Edouard Vaillant, Villejuif 94805, France; Centre de Recherche des Cordeliers, Equipe labellisée par la Ligue contre le cancer, Université de Paris, Sorbonne Université, INSERM U1138, Institut Universitaire de France, 15 Rue de l'École de Médecine, Paris 75006, France; Institute of Physiology II, University of Münster, Münster 48149, Germany; Division of Cardiology and Center for Cardiovascular Research, Washington University School of Medicine, St. Louis, MO, USA; Department of Cell Biology and Molecular Medicine, Rutgers New Jersey Medical School, Newark, NJ, USA; Department of Biochemistry, Chungbuk National University, Cheongju, South Korea; Department of Cell Biology and Molecular Medicine, Rutgers New Jersey Medical School, Newark, NJ, USA; Department of Cardiology, Medical University of Graz, Auenbruggerplatz 15, Graz 8036, Austria; Department of Cardiology, Dongzhimen Hospital, Beijing University of Chinese Medicine, Beijing, China; Metabolomics and Cell Biology Platforms, Institut Gustave Roussy, 114 Rue Edouard Vaillant, Villejuif 94805, France; Centre de Recherche des Cordeliers, Equipe labellisée par la Ligue contre le cancer, Université de Paris, Sorbonne Université, INSERM U1138, Institut Universitaire de France, 15 Rue de l'École de Médecine, Paris 75006, France; BioTechMed-Graz, Mozartgasse 12/I, Graz 8010, Austria; Division of Cell Biology, Histology and Embryology, Gottfried Schatz Research Center for Cell Signaling, Metabolism and Aging, Medical University of Graz, Graz 8010, Austria; Division of Molecular Biology and Biochemistry, Gottfried Schatz Research Center for Cell Signaling, Metabolism and Aging, Medical University of Graz, 8010 Graz, Austria; Department of Cell Biology and Molecular Medicine, Rutgers New Jersey Medical School, Newark, NJ, USA; Division of Cardiology and Center for Cardiovascular Research, Washington University School of Medicine, St. Louis, MO, USA; John Cochran Veterans Affairs Medical Center, St. Louis, MO, USA; Institute of Physiology II, University of Münster, Münster 48149, Germany; Department of Internal Medicine, Centro Hospitalar Universitário de São João, 4200-319 Porto, Portugal; Department of Surgery and Physiology, Faculty of Medicine of the University of Porto, Cardiovascular R&D Center (UnIC@RISE), 4200-319 Porto, Portugal; Metabolomics and Cell Biology Platforms, Institut Gustave Roussy, 114 Rue Edouard Vaillant, Villejuif 94805, France; Centre de Recherche des Cordeliers, Equipe labellisée par la Ligue contre le cancer, Université de Paris, Sorbonne Université, INSERM U1138, Institut Universitaire de France, 15 Rue de l'École de Médecine, Paris 75006, France; Pôle de Biologie, Hôpital Européen Georges Pompidou, AP-HP, 20 Rue Leblanc, Paris 75015, France; Department of Cardiology, Medical University of Graz, Auenbruggerplatz 15, Graz 8036, Austria; BioTechMed-Graz, Mozartgasse 12/I, Graz 8010, Austria; Faculty of Medicine, University of Maribor, Taborska ulica 8, Maribor 2000, Slovenia

**Keywords:** Metabolic cardiomyopathy, HFpEF, Obesity, Autophagy, Mitophagy, NAD^+^ metabolism

Metabolic cardiomyopathy is a major complication of obesity that often progresses to clinically evident heart failure with preserved ejection fraction (HFpEF).^1^ This predominant cardiometabolic form of HFpEF is a burgeoning public health problem with limited evidence-based therapies.^2^ In this regard, we previously showed that clinical HFpEF is associated with low cardiac levels of the metabolic cofactor nicotinamide adenine dinucleotide (NAD^+^) and that oral supplementation of nicotinamide (NAM), an NAD^+^ precursor, improves preclinical HFpEF in several rodent models.^3^ However, NAD^+^ is a pleiotropic molecule and its mode of action in HFpEF remains elusive. Because autophagy is an NAD^+^-stimulated mechanism that is essential for maintaining cellular homeostasis and mitochondrial health, particularly in long-lived post-mitotic cardiomyocytes under metabolic stress,^4^ we speculated that an increase of autophagic flux might contribute to the efficacy of NAM supplementation against metabolic cardiomyopathy and associated HFpEF.

To determine whether NAD^+^ supplementation stimulates cardiac autophagy in cardiometabolic HFpEF, we assessed autophagy markers at both the mRNA and protein levels in NAM-fed ZSF1 obese rats, a model of cardiometabolic syndrome and HFpEF. At variance with our previous report,^3^ we administered a 40% lower and, hence, a more clinically feasible dose of NAM (0.3% w/v in the drinking water), which increased cardiac NAD^+^ by 30 ± 11% (mean ± SD) in treated vs. control rats. Moderate elevation of NAD^+^ was not associated with a significant difference in ejection fraction (*Figure 1A*), but efficiently improved cardinal signs of HFpEF. Specifically, NAM reduced cardiac hypertrophy and diastolic dysfunction, as indicated by lower echocardiography-derived left ventricular (LV) mass, LV remodelling and functional indices as well as pulmonary congestion, as measured by tibia length-normalized lung weight (*Figure 1A*). These cardioprotective effects of NAM coincided with increased expression of several mRNA species relevant to the autophagic-lysosomal pathway in the heart (*Figure 1B*). Consistently, NAM stimulated the autophagy-associated lipidation of microtubule-associated protein 1A/1B-light chain 3B (LC3B), leading to an increase in LC3B-II, while reducing the abundance of the autophagic substrate sequestosome 1 (SQSTM1, commonly known as p62) to the levels of lean controls (*Figure 1B*). Collectively, these findings suggest that the beneficial effects of NAM in obesity-related HFpEF coincide with restored cardiac autophagy. Notably, NAM also increased selective degradation of cardiac mitochondria detectable in mice expressing the mitophagy biosensor Mito-Keima mice^5^ (*Figure 1B*), suggesting that NAM might ameliorate cardiac function, at least in part, by improved mitochondrial quality control.

**Figure 1:**
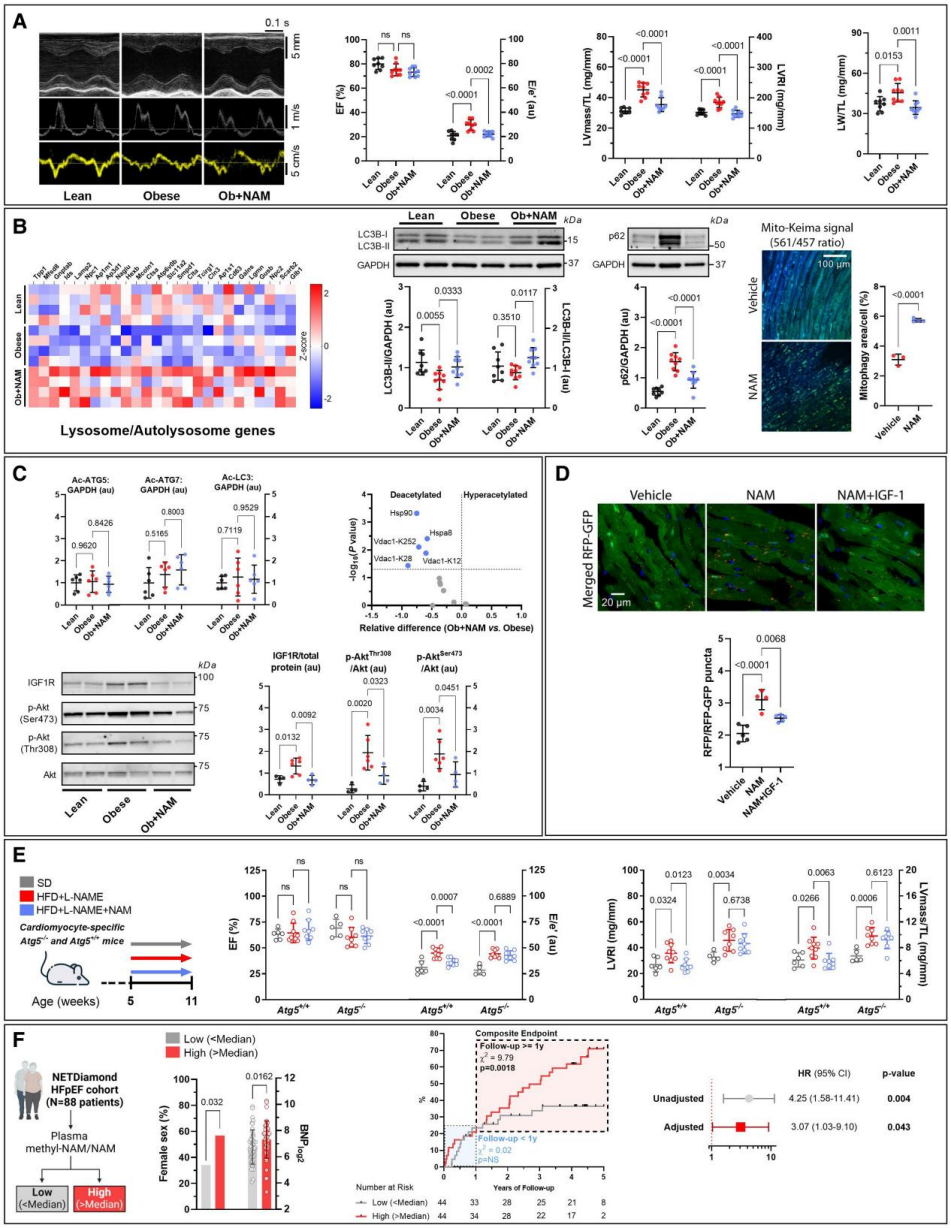
Nicotinamide improves cardiometabolic HFpEF through autophagy activation. (*A*) Representative echocardiography tracings: M-mode (top), pulsed-wave Doppler (middle), and tissue Doppler (bottom) from 20-week-old ZSF1 lean and obese rats, treated or not with 0.3% v/w nicotinamide (NAM) in the drinking water for 12 weeks (Ob + NAM vs. Obese, respectively). EF, ejection fraction. Left ventricular mass (LVmass) normalized to tibia length (TL). Ratio of peak early Doppler transmitral flow velocity (E) to myocardial tissue Doppler velocity (e′). Lung weight normalized to tibia length (LW/TL) (*n* = 8–9 rats/group). (*B*) Heatmap of cardiac expression levels (red = high, blue = low) of differentially regulated genes involved in the autolysosome-lysosome KEGG pathway (left), (*n* = 4 rats/group). Representative Western blots and quantification (middle) of autophagy markers, including LC3B-II expression, LC3B-II-to-LC3B-I ratio, and the autophagy substrate p62 (normalized to GAPDH) in the hearts of ZSF1 lean, obese, and NAM-treated obese rats (*n* = 8/9/9 rats, respectively). Representative confocal images of myocardial sections from adult Mito-Keima reporter mice (3-month-old) treated with the mouse equivalent dose of NAM (0.5% v/w in the drinking water for two weeks), with quantitative assessment of the positive ratiometric area of Mito-Keima fluorescence (561 nm/467 nm excitation), indicating mitophagy (right). Values were obtained from 30 different pictures per sample (*n* = 3–4 mice/group). (*C*) Immunoblot-based quantification of autophagy proteins acetylation (normalized to GAPDH) in the hearts of ZSF1 lean, obese, and NAM-treated obese rats (*n* = 6 rats/group), along with a Volcano plot depiction of the relative difference in detectable autophagy-related protein acetylation in NAM-treated vs. control obese ZSF1 rats (top), (*n* = 4/3 rats, respectively). Representative Western blots and quantification of insulin-like growth factor-1 receptor (IGF-1R) expression (normalized to total protein content detected by Ponceau S staining), and Akt phosphorylation normalized to total Akt expression in the hearts of ZSF1 lean, obese, and NAM-treated obese rats (bottom), (*n* = 4/6/4 rats, respectively). (*D*) Representative confocal images of myocardial sections from adult CAG-RFP-GFP-LC3 transgenic mice (3-month-old) treated with NAM (0.5% v/w in the drinking water for two weeks) and IGF-1 (200 µg/kg i.p., 30 min before sacrifice) or vehicle, with quantitative assessment of the ratio of autolysosomes (punctate RFP signal) to autophagosomes (punctate RFP-GFP signal (*n* = 4–5 mice/group). (*E*) Schematic representation of NAM feeding protocol to 5-week-old cardiomyocyte-specific *Atg5*-deficient mice (*Atg5^−/−^*), generated by crossing *Atg5^flox/flox^* mice with knock-in mice expressing Cre^+^ recombinase driven by the cardiomyocyte-specific *MLC2a* gene encoding α-myosin light chain promoter (*MLC2a-Cre^+^*). *Atg5^−/−^* mice and their control littermates (*Atg5^+/+^*) were fed a standard diet (SD; Sniff, #V1534) or a combination of high-fat diet (HFD; 45% kcal from fat, lard; Sniff, cat. EF R/M #D12451 modified) and the nitric oxide synthase inhibitor L-NAME (0.5 g/L in the drinking water) in the presence or absence of NAM (0.5% v/w in the drinking water for 6 weeks). EF, E/e′ ratio, LVRI, LVmass/TL. (*n* = 5–9 mice/group) (*F*) NAM and methyl-NAM levels were measured in participants of the NETDiamond prospective HFpEF cohort (*N* = 88). Participants were stratified by the median of methyl-NAM/NAM ratio, a proxy of NAM depletion, and compared across demographic, analytical, and echocardiographic parameters; only statistically significant differences [sex and B-type natriuretic peptides (BNP)] are reported. Time-to-first-event analysis for the composite endpoint of cardiovascular death or HF progression (i.e. HF hospitalization, acute HF episodes, or diuretic intensification) was conducted using Kaplan–Meier curves and Cox proportional-hazards models. The analysis was stratified with split time at 1 year of follow-up due to a significant time-dependent interaction, indicating a violation of the proportional hazards assumption (*P* = 0.036). Hazard ratios (HR) with 95% confidence intervals (CI) for follow-up ≥1 year are presented for unadjusted and adjusted models, with covariates including age, sex, creatinine, and BNP levels. Data are presented as means ± SD with subject-level data superimposed as individual points. *P* values were calculated by ANOVA with Dunnett’s *post hoc* test (*A*–*D*), Student’s *t*-test (*B*, right), or two-way ANOVA with Dunnett’s *post hoc* test (*E*). In (*F*), *P* values were calculated by Mann–Whitney test, χ^2^ test, Log-Rank test, or Cox proportional-hazards model, respectively. Akt, Akt serine/threonine kinase 1; Hsp90, heat shock protein 90; Hspa8, heat shock cognate 71 kDa protein; LC3, microtubule-associated protein 1A/1B-light chain 3; p62, ubiquitin-binding protein p62; ns, non-significant; Vdac1, voltage-dependent anion-selective channel protein 1.

Mechanistically, NAD^+^ can activate autophagy through sirtuin-1, which deacetylates essential autophagy proteins, such as ATG5, ATG7, and LC3.^6^ However, none of these autophagy proteins showed altered acetylation in obese rats at baseline or after NAM treatment (*Figure 1C*). Therefore, we performed a whole cardiac acetylome analysis, which revealed modest NAM-induced deacetylation of a limited number of proteins linked to autophagy, but none of the essential autophagy machinery proteins exhibited detectable acetylation in either of the groups (*Figure 1C*). Thus, we examined the cardiac transcriptome to unveil the molecular basis for autophagy activation in NAM-treated rats. Interestingly, the insulin/IGF-1 pathway, a major nutrient-sensing and inhibitory pathway of autophagy in the heart,^7^ emerged amongst the top hits in an unbiased gene-set enrichment analysis (Benjamini–Hochberg-adjusted *P* = 0.006; KEGG). Accordingly, NAM reduced the expression of IGF-1 receptor to the levels of lean control hearts, and diminished the activity of IGF-1 signalling, as indicated by reduced phosphorylation of the downstream effector serine/threonine-protein kinase Akt at Thr^308^ and Ser^473^ (*Figure 1C*). More importantly, IGF-1 administration reduced the capacity of NAM to induce autophagic flux in cardiomyocytes (*Figure 1D*), as evaluated in a tandem-tagged autophagy reporter mouse model (*CAG-RFP-EGFP-LC3*). Together, these findings demonstrate that attenuated IGF-1 signalling, rather than deacetylation of autophagic machinery proteins, underlies the pro-autophagic activity of NAM in the heart.

Next, we examined whether autophagy is causally required for the anti-HFpEF effects of NAM using *Atg5*-deficient (*Atg5^−/−^*) mice, which lack constitutive autophagy specifically in cardiomyocytes and show no echocardiography-detectable systolic and diastolic dysfunction until the age of 15 weeks.^5^

Therefore, we used 5-week-old *Atg5^−/−^* mice and their control littermates (*Atg5^+/+^*) that were subjected to a ‘two-hit’ HFpEF model using a high-fat diet (HFD) and the nitric oxide synthase inhibitor N[ω]-nitro-l-arginine methyl ester (L-NAME).^8^ After 6 weeks of combined administration of HFD and L-NAME, both *Atg5^−/−^* and *Atg5^+/+^* mice exhibited preserved ejection fraction, but developed diastolic dysfunction, LV remodelling and hypertrophy (*Figure 1E*). However, as compared to *Atg5^+/+^* mice, we found exaggerated LV hypertrophy in *Atg5^−/−^* mice upon HFD + L-NAME administration (*Figure 1E*). NAM significantly improved diastolic dysfunction and LV remodelling in HFD + L-NAME-fed *Atg5^+/+^* mice but failed to exert similar cardioprotective effects in *Atg5^−/−^* mice (*Figure 1E*). Notably, NAM similarly reduced body weight gain in *Atg5^−/−^* and *Atg5^+/+^* mice (not shown), supporting the cardiac-specific action of NAM.

Finally, we evaluated the translational potential of our findings in a prospective cohort of patients with HFpEF (NETDiamond study).^9^ We quantified circulating NAM and its degradation metabolite, methyl-NAM. HFpEF patients with a higher methyl-NAM/NAM ratio, denoting reduced bioavailability of NAM for NAD^+^ biosynthesis, were more likely to be females and had elevated B-type natriuretic peptides (BNP), but did not exhibit any significant difference in other classical cardiovascular comorbidities, compared to those with a lower methyl-NAM/NAM ratio (*Figure 1F*). More importantly, a higher methyl-NAM/NAM ratio was associated with a significantly higher risk of adverse outcomes (a composite of cardiovascular death, HF hospitalization, acute HF episode, or diuretic intensification) in the stratified analysis after one year of follow-up (*Figure 1F*). This association remained significant after adjusting for age, sex, creatinine, and BNP levels (*Figure 1F*).

This study has some limitations. Although the animal models of cardiometabolic HFpEF used here exhibit similar metabolic and haemodynamic stress, they differ in their aetiology. ZSF1 obese rats develop hyperphagia-induced obesity and spontaneous hypertension, while HFD + L-NAME mice become obese and hypertensive in response to hypercaloric diet and constitutive nitric oxide synthase inhibition, respectively. Additionally, because *Atg5^−/−^* mice develop cardiac dilation early in life, we administered HFD and L-NAME for a short period at a young age. We also acknowledge that the methyl-NAM/NAM ratio measured in plasma only constitutes a proxy of NAD^+^ homeostasis, knowing that NAD^+^ itself is undetectable in plasma.

Notwithstanding these limitations, we concluded that reinstating cardiac autophagy through reduced IGF-1 signalling is essential for NAM to ameliorate cardiometabolic HFpEF. Our findings reaffirm the therapeutic potential of stimulating autophagy through down-regulation of excessive nutrient signalling in HFpEF, and align with the proposed mechanism of action for SGLT2 inhibitors,^10^ the first evidence-based treatment for HFpEF. Hence, clinical studies are warranted to determine whether activating autophagy through NAD^+^ precursors supplementation will constitute an effective strategy to treat HFpEF in patients. For this, systematic dose–response studies must determine whether the reported lower NAM dose herein, corresponding to 1.5 g per day for an average adult weighing 70 kg, will be clinically efficient.

## Declarations

### Data Availability

Data and materials supporting the results or analyses presented in this paper are available on reasonable request to the corresponding authors. The data underlying this article were not provided by a third party.

### Pre-registered Clinical Trial Number

None supplied.
